# Downregulation of type I collagen expression in the Achilles tendon by dexamethasone: a controlled laboratory study

**DOI:** 10.1186/s13018-020-01602-z

**Published:** 2020-02-24

**Authors:** Zilu Ge, Hong Tang, Wan Chen, Yunjiao Wang, Chengsong Yuan, Xu Tao, Binghua Zhou, Kanglai Tang

**Affiliations:** Department of Orthopedics/Sports Medicine Center, State Key Laboratory of Trauma, Burn and Combined Injury, Southwest Hospital, Third Military Medical University (Army Medical University), Gaotanyan Street. 30, Shapingba District, Chongqing, 400038 China

**Keywords:** Achilles tendon, Dexamethasone, Human, Rats, Type I collagen

## Abstract

**Background:**

Spontaneous Achilles tendon rupture associated with long-term dexamethasone (Dex) use has been reported. However, few studies have investigated the potential mechanism. The aim of this study was to evaluate the effects of oral Dex on type I collagen in humans and rats and its association with tendon rupture.

**Methods:**

First, six Achilles tendons from patients who received long-term Dex treatment, and another six normal tendons were harvested for histological evaluation. Secondly, 8-week-old rats (*n* = 72) were randomly assigned to a Dex group or a control group. Type I collagen was studied at the mechanical, histological, and molecular levels after 3 and 5 weeks. Tenocytes isolated from normal human and rat tendon were used to investigate the effect of Dex on cellular scale.

**Results:**

Histological analysis of human and rat tendon tissue revealed an irregular, disordered arrangement of type I collagen in the Dex group compared with the control group. In addition, In the Dex+ group, type I collagen expression decreased in comparison with the Dex− group in both human and rat tenocytes. The mechanical strength of tendons was significantly reduced in the Dex group (68.87 ± 11.07 N) in comparison with the control group (81.46 ± 7.62 N, *P* = 0.013) after 5 weeks. Tendons in the Dex group were shorter with smaller cross-sectional areas (10.71 ± 0.34 mm^2^, 1.44 ± 0.22 mm^2^, respectively) after 5 weeks than those in the control group (11.13 ± 0.50 mm^2^, *P* = 0.050, 2.74 ± 0.34 mm^2^, *P* < 0.001, respectively).

**Conclusions:**

This finding suggests long-term use of Dex that decreases the expression of type I collagen at molecular and tissue levels both in human and rat Achilles tendons. Furthermore, Dex decreases the mechanical strength of the tendon, thereby increasing the risk of Achilles tendon rupture.

## Introduction

Since the first use of glucocorticoids (GC) in September 1948, at the Mayo Clinic, to treat a patient with rheumatoid arthritis (RA) [1], they have been extensively used to treat osteoarthritis, tendinopathy, inflammatory arthritis, and degenerative spine disease. In addition to rest, oral nonsteroidal anti-inflammatory drugs, specific stretching, and strengthening exercises, GC treatment is commonly prescribed for Achilles tendon diseases [2]. Although it is generally accepted that local injections of GCs temporarily alleviate local inflammation and pain, the adverse effects and long-term functional consequences have limited their use [3]. The local administration of GCs has significant negative effects on tendons, increasing the risk of tendon rupture, impairing tendon healing, and leading to poorer long-term outcomes [4]. However, only a few studies have reported a link between oral GCs and the risk of Achilles tendon rupture [5, 6]. Considering occasional case reports of patients receiving long-term GC treatment due to systematic diseases or autoimmune disorders, who experienced spontaneous Achilles tendon rupture [7–9], it is important to determine the exact mechanism by which the Achilles tendon is weakened by GC.

The strength and stability of the Achilles tendon are significantly affected by collagen, which is the main component of the tendon. The amount of collagen and its spatial arrangement are important determinants of the mechanical properties of tendons [10]. In particular, type I collagen accounts for approximately 85–95% of the dry weight of an Achilles tendon, making it the foremost factor in the stabilization of the tendon [11]. Type I collagen, as well as elastin fibers and many other extracellular matrix components (cytokines, enzymes, and glycosaminoglycans), is produced by tenocytes [12]. Any factor contributing to collagen denaturation, decrease, or change in arrangement may lead to the weakening or rupture of the Achilles tendon.

Researchers have suspected that spontaneous and low-impact ruptures of the Achilles tendon are associated with GC treatment. This can be explained by a disruption in the structure of type I collagen by GC. A series of animal experiments support this hypothesis. Under different doses of GCs, the level of type I collagen decreases, to a certain extent, in the rotator cuff tendon of rats [13]. An in vitro analysis of tendon cells has shown that GCs reduce cell viability, cell proliferation, and collagen synthesis [14]. However, few studies have directly demonstrated the effect of oral GCs on type I collagen in humans. We hypothesized that long-term use of dexamethasone (Dex) could decreased the expression of type I collagen in the Achilles tendon. Therefore, we used samples harvested from patients who underwent long-term Dex treatment to evaluate its effect on type I collagen in human Achilles tendons. To avoid any interference from the clinic, we further confirmed the results using mechanical, histological, and molecular analysis of rat tendons.

## Methods

### Achilles tendon samples

All experiments and study protocols, including sample acquisition and analysis, were approved by the institutional review board of the Human Research Ethics Committee of Army Military Medical University (KY201838). Human Achilles tendon specimens were obtained from patients in the Department of Orthopaedic Sports Medicine Center, Southwest Hospital, Army Military Medical University, Chongqing, China, after obtaining their written informed consent. Patients who met the inclusion and exclusion criteria were involved in this study. In general, the inclusion criteria for Dex group included patients who suffered Achilles tendon rupture and had a history of long-term Dex treatment for RA, and the cumulative duration of Dex treatment was more than 3 months. The inclusion criteria for control group included patients who suffered Achilles tendon rupture due to acute trauma but had no treatment history of any glucocorticoids. For both groups, patients aged below 18 or above 50 years, diagnosis of Achilles tendinopathy, type 1 or 2 diabetes, hypertension and hyperlipidemia, Achilles tendon infection, smoke and alcohol addiction, previous injury or surgery on Achilles tendon, and overuse of the Achilles tendon would lead to exclusion. After ensuring they met the inclusion and exclusion criteria, six patients were included in the Dex group, and six patients were served as the control group. Patients’ demographics, including age (Dex group, 37.3 ± 1.6; control group, 35.3 ± 2.0; *P* = 0.451), body mass index (Dex group, 22.8 ± 0.8; control group, 23.5 ± 0.9; *P* = 0.586), and gender (Dex group, 50%; control group, 50%), showed no significant differences between the Dex and control groups.

All samples were collected during tendon repair surgery. After removing the peritendinous tissue, 2 × 2-mm sample near the edge of torn Achilles tendon was harvested from each patient in the Dex and control group. The tissue was immediately placed in formaldehyde fixation fluid to prepare it for histological staining. Another 2 × 2-mm tissue sample was harvested from each patient in the control group and was placed in sterile phosphate-buffered saline (PBS) for cellular experiment.

**Fig. 1:**
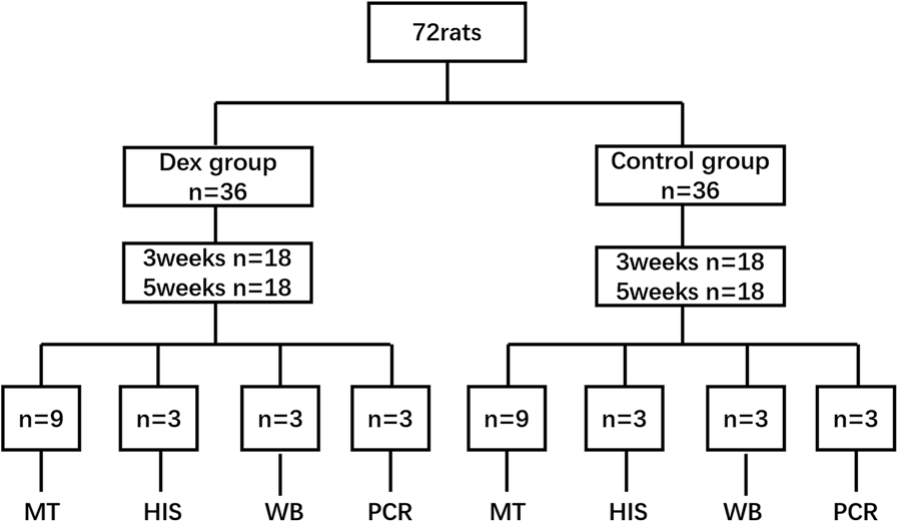
Flowchart of animal experiment. MT, mechanical testing; HIS, histology; WB, western blotting; qRT-PCR, quantitative real-time polymerase chain reaction

For the animal experiment, 72 male, 8-week-old Sprague–Dawley rats, weighing approximately 200 g, were stratified by body weight and then randomized into four groups (18 animals per group, 9 for the mechanical test, 3 for western blotting, 3 for PCR, and 3 for immunostaining (Fig. 1). Owing to the poor solubility of Dex, 3% dimethyl sulfoxide (DMSO) was added [15]. The animals that were randomly selected to receive Dex and DMSO (Dex group) were treated by intraperitoneal injection into the abdomen with 10 mg/mL/kg [16] once daily for 3 and 5 weeks. Animals in the control group were injected with saline and DMSO at the same dose. At the endpoint, animals were euthanized under inhalation anesthesia with isoflurane, and the left tendons were used for all experiments. Cells isolated from tendons of the control group were used to evaluate the impact of Dex on rat tenocytes. All animals were kept in individual cages under the same conditions of feeding and environment and without activity restriction.

### Isolation and culture of tenocytes

Isolation and culture of human and rat tenocytes were performed as per our previous description [17, 18]. Briefly, after collecting the mid-portion of the Achilles tendon, the peritendinous connective tissue was carefully removed. The remaining tissue was then placed in PBS and finely diced before being digested in type I collagenase (3 mg/mL Sigma–Aldrich, St. Louis, MO, USA) for 2.5 h at 37 °C on a rocking bed set at 200 rpm. A single-cell suspension was yielded using a 70-μm cell strainer (Becton Dickinson, Franklin Lakes, NJ, USA). After washing in PBS, the released cells were centrifuged at 1500 rpm for 5 min and were then resuspended in Dulbecco’s modified Eagle’s medium (DMEM) (Gibco, Carlsbad, CA, USA) containing 100 U/mL penicillin, 100 mg/mL streptomycin, 10% fetal bovine serum, and 2 mmol/L l-glutamine (all from Invitrogen, Carlsbad, CA, USA). The isolated cells were plated at 37 °C in 5% CO_2_ for 2 days to form colonies and were then washed twice in PBS to remove nonadherent cells. On day 7 of culture, cells were trypsinized with trypsin-EDTA solution (Sigma-Aldrich), combined together, and were seeded onto plates as passage 0 (P0) cells. All subsequent experiments were conducted using cells at P3. Tenocytes were seeded onto 6-well plates with a density of 6 × 10^4^ cells/well and 10-cm-diameter petri dishes for qRT-PCR and protein extraction, respectively. During the culture period, DMEM used for culturing cells in the Dex+ group was supplemented with 1 μM Dex (Sigma–Aldrich) and dissolved in DMSO, and the Dex− group were cultured in DMEM supplemented with DMSO only. All media were changed every 3 days.

### Tenocyte identification

Human and rat tenocyte identification was carried out based on the immunoreactivity of the cells for collagen I [19] and vimentin [20] and negative immunoreactivity for collagen III [21]. The primary cultured tenocytes were used for immunostaining. Briefly, tenocytes were cultured on coverslips in a 12-well plate at a density of 1–2 × 10^5^ cells/ml for 24 h. The cells were then fixed in 4% formaldehyde for 30 min at room temperature. After washing, normal goat serum (5%) was added to the coverslips and incubated for 30 min at room temperature to block non-specific binding sites. Cells were incubated with diluted primary antibody in 5% PBS Tween 20 (PBST) overnight at 4 °C. The cells were then incubated with secondary antibody (fluorescein-isothiocyanate-labeled goat anti-mouse, Santa Cruz, Dallas, USA) at room temperature in the dark with 5% BSA for 1 h. After incubating with 0.5 μg/mL DAPI (Beyotime, Shanghai, China) for 5 min, the coverslips were mounted using mounting medium. Five areas were randomly selected and observed under × 200 magnification using a confocal laser scanning microscope (TCS2NT; Leica, Wetzlar, Germany).

The primary antibodies used in the human tenocytes identification were as follows: monoclonal mouse anti-human collagen I (Sigma-Aldrich, Munich, Germany), mouse anti-human collagen III (Sigma-Aldrich), and mouse anti-human vimentin (Sigma-Aldrich). The primary antibodies used in the rat tenocyte identification were as follows: rabbit anti-rat collagen I, rabbit anti-rat collagen III, and rabbit anti-rat vimentin (all from Abcam).

### Immunohistochemical staining

Serial sagittal paraffin sections were prepared from the Achilles tendons as previously described [22]. Briefly, all samples (including rat and human) for histology were fixed in 4% buffered formalin. After embedding in paraffin, the tissue was cut into 4-μm sections. The sections were blocked with H_2_O_2_ and methanol for 15 min under dark light after deparaffinization. Sections were then incubated with pepcase and tryptase for 30 min at 37 °C for antigen retrieval. After incubating with goat serum for 30 min, sections were incubated with anti-collagen type I (1:500) (Abcam) in 5% BSA in PBST overnight at 4 °C. Sections were then incubated with enzyme-conjugated secondary antibody for 2 h at room temperature. After being dehydrated and mounted, five areas obtained from each sample were randomly selected, and an OLYMPUS EX-51 light microscope (Tokyo, Japan) was used to observe samples at × 40 and × 200 magnification. The whole areas were evaluated by the Bonar score system [23]. The score of the intact group was defined as 12 points, and the average optimal density (AOD) of type I collagen was calculated by ImageJ.

### qRT-PCR

Quantitative real-time polymerase chain reaction (qRT-PCR) was used to evaluate type I collagen mRNA levels. After extraction of RNA from the cells or tissues by TRIzol (TaKaRa, Kusatsu, Japan), and synthesizing cDNA (TaKaRa), qRT-PCR was performed using a SYBR Green RT-PCR Kit (TaKaRa) and ABI Prism 7900 Sequence Detection System (PE Applied Biosystems, Foster City, CA, USA). The expression levels were calculated relative to the expression of glyceraldehyde 3-phosphate dehydrogenase (*GAPDH*). Primer sequences are shown in Table 1.

**Table 1:** Sequences of primers used for qRT-PCR

Gene	Species	Forward primer (5′→3′)	Reverse primer (3′→5′)
Col I	Human	TGGTGAGACGTGGAAACCTG	CTTGGGTCCCTCGACTCCTA
Col I	Rats	TGGCAAAGAAGGCGGCAAAGG	AGGAGCACCAGCAGGACCATC
GAPDH		TGACTTCAACAGCAACTC	TGTAGCCATATTCATTGTCA

### Protein extraction and western blotting

After tissue was cut into pieces and cells were scraped, they were homogenized in lysis buffer (50 mmol/L Tris-HCl, pH 8.0, 1 mmol/L EDTA, 1% Triton X-100, 0.5% sodium deoxycholate, 0.1% sodium dodecyl sulphate (SDS), 150 mmol/L NaCl) containing proteinase inhibitors (Thermo Fisher Scientific Inc., Rockford, IL, USA). A BCA protein assay kit (Thermo Fisher Scientific Inc., Rockford, IL, USA) was used for protein concentration measurement. Protein samples (30 μg/lane) were resolved by SDS-polyacrylamide gel electrophoresis and then transferred onto polyvinylidene difluoride membranes. The membranes were blocked with 0.1% TBS-Tween containing 5% non-fat milk for 1 h at room temperature, then incubated sequentially with primary antibody (anti-collagen I (Abcam)) and secondary antibody (goat anti-rabbit IgG (H&L)-HRP conjugated (Proteintech, 1:3000)). The results were visualized, and images were captured using a LiCoR Odyssey Imager (LI-COR Biosciences, Lincoln, NE, USA).

### Mechanical testing

A high-precision caliper was used to record the length, width, and thickness of the tendon. Subsequently, the Achilles tendon, with half of the muscle, was fixed in a mechanical testing machine to determine the load to failure (N) and the elastic modulus (MPa) [24, 25]. After the gastrocnemius was frozen in liquid nitrogen [26], it was fastened to the machine (Regerl, China). Tendons were tested one by one under a constant displacement rate of 50 mm/min. Ringer’s solution was used to moisten the tendon during the test. Data on load to failure (N) were obtained from the load-elongation graph. Elastic modulus (MPa) was calculated by the linear portion of the graph and was correlated with strain and cross-sectional area.

### Statistical analysis

A 5% significance level and an 80% power were set based on previous studies [16]. The sample size was 10 rats per group in the mechanical test. Similar experiments in previous studies have used 7–9 animals in mechanical tests and 3–10 animals for histological outcomes. Therefore, we believe that this sample size was reasonable. Comparisons of mechanical properties among groups were performed using independent sample *t* tests after normality testing and homogeneity testing of variance. Differences with *P* < 0.05 were considered statistically significant. All analyses were performed using SPSS version 22.0 (IBM Corp., Armonk, NY, USA).

## Results

### Dex downregulates type I collagen expression in human Achilles tendons

**Fig. 2:**
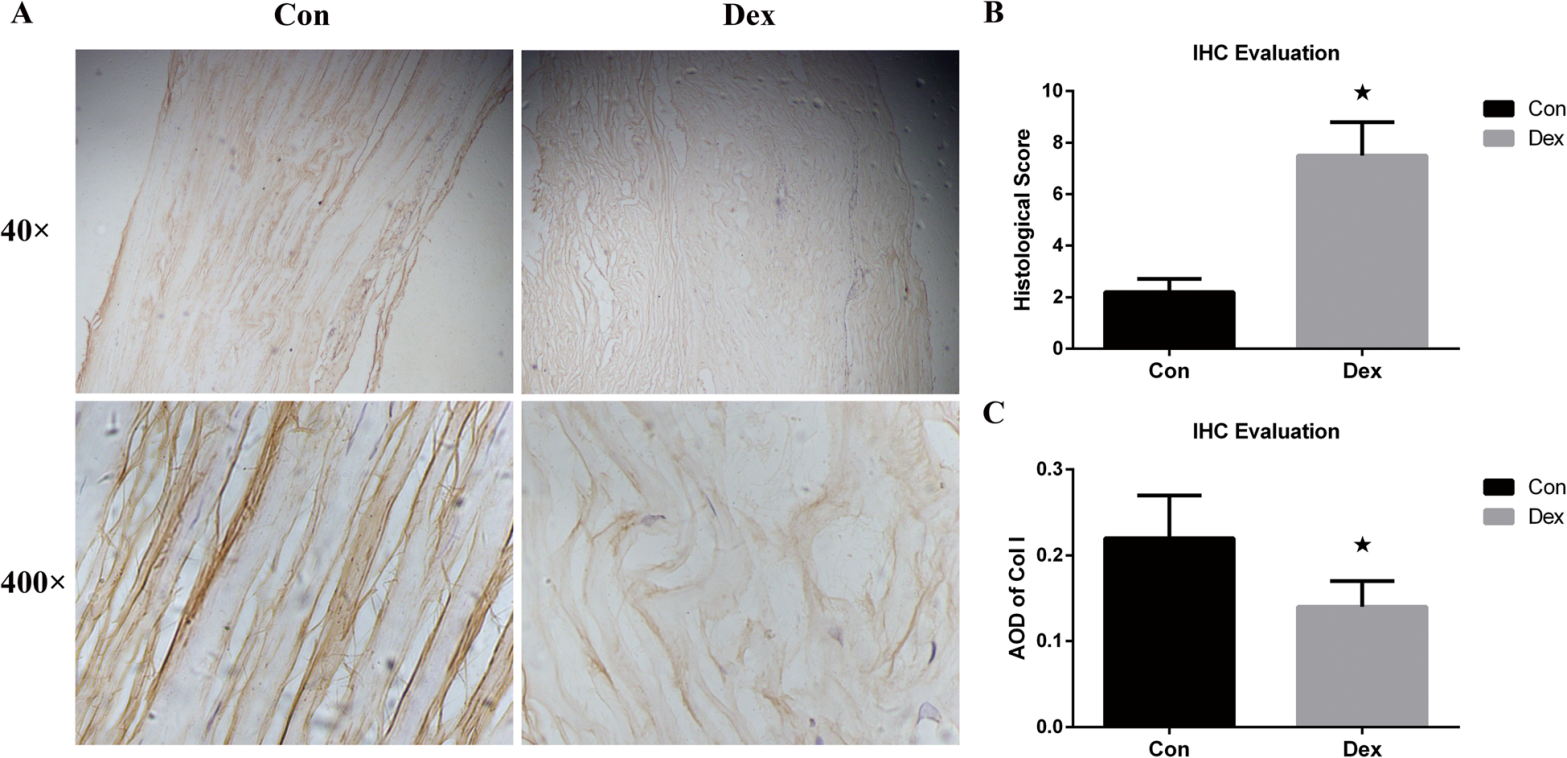
**a** Histology of human Achilles tendons. The yellow fasciculate bands represent type I collagen. The Dex group, receiving long-term Dex treatment, have irregular and curled collagen type I. **b** The histological score of immunohistochemical evaluation (IHC). **c** The average optimal density (AOD) of type I collagen expressed in human Achilles tendons

In a comparison between tissue harvested from the ruptured Achilles tendon of patients who had a history of long-term Dex use and from patients who suffered from acute trauma, we observed a distinct difference in type I collagen (Fig. 2a). The quality and density of type I collagen in Achilles tendons that ruptured by acute trauma were basically normal. Collagen was arranged regularly and was thicker than the collagen in the Dex group. The ruptured human Achilles tendon induced by Dex treatment showed collagen attenuation, with a highly irregular arrangement and a disordered and curled appearance in the whole field of vision. The histological score of tissue and AOD of type I collagen was showed in Fig. 2b and c.

**Fig. 3:**
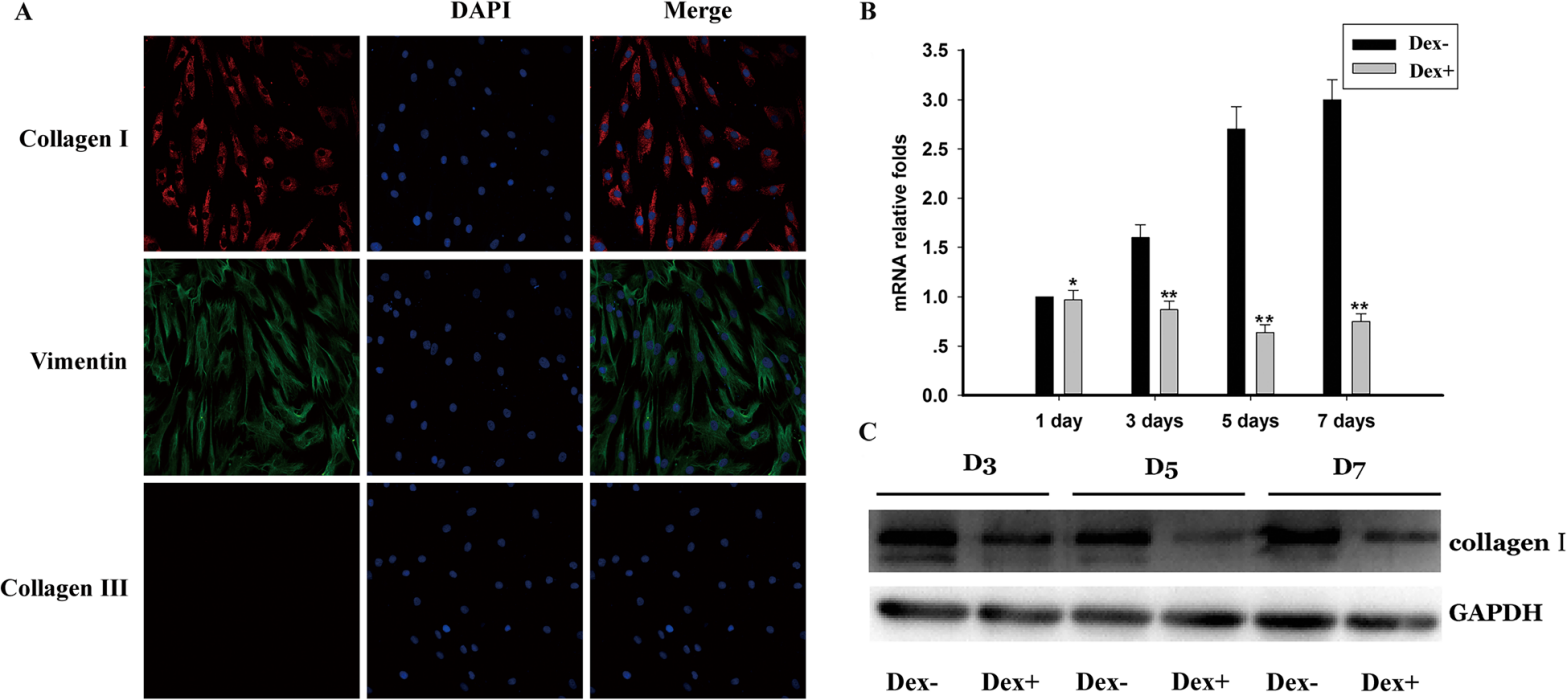
**a** Identification of human tenocytes. Collagen type I and vimentin were positively expressed, and Collagen type III was negatively expressed. **b** mRNA expression of type I collagen in human Achilles tenocytes. The black and gray bars represent the Dex− and Dex+ groups, respectively. The asterisk represents a significant change between the two groups. **c** Protein expression of type I collagen in human Achilles tenocytes. Relative expression levels were normalized to *GAPDH*

To evaluate our hypothesis regarding the role of type I collagen in tendon rupture at the cellular level, we isolated human tenocytes from tissues damaged by trauma and cultured them in DMEM with and without Dex. The human tendon cells were fusiform-shaped, as shown in Fig. 3a, and qRT-PCR analysis showed that there were no significant changes in type I collagen expression after treatment with Dex for 1 day. The expression level gradually increased in the Dex− group. However, unlike the upward trend observed in the Dex− group, the expression of type I collagen decreased gradually after 3 and 5 days and increased slightly at 7 days in the Dex treatment group. Expression levels at all time points were significantly lower than those in the Dex− group, and the gap increased over time (Fig. 3b). The western blotting results showed the same trend (Fig. 3c).

### Dex downregulates type I collagen expression in rat Achilles tendons

**Fig. 4:**
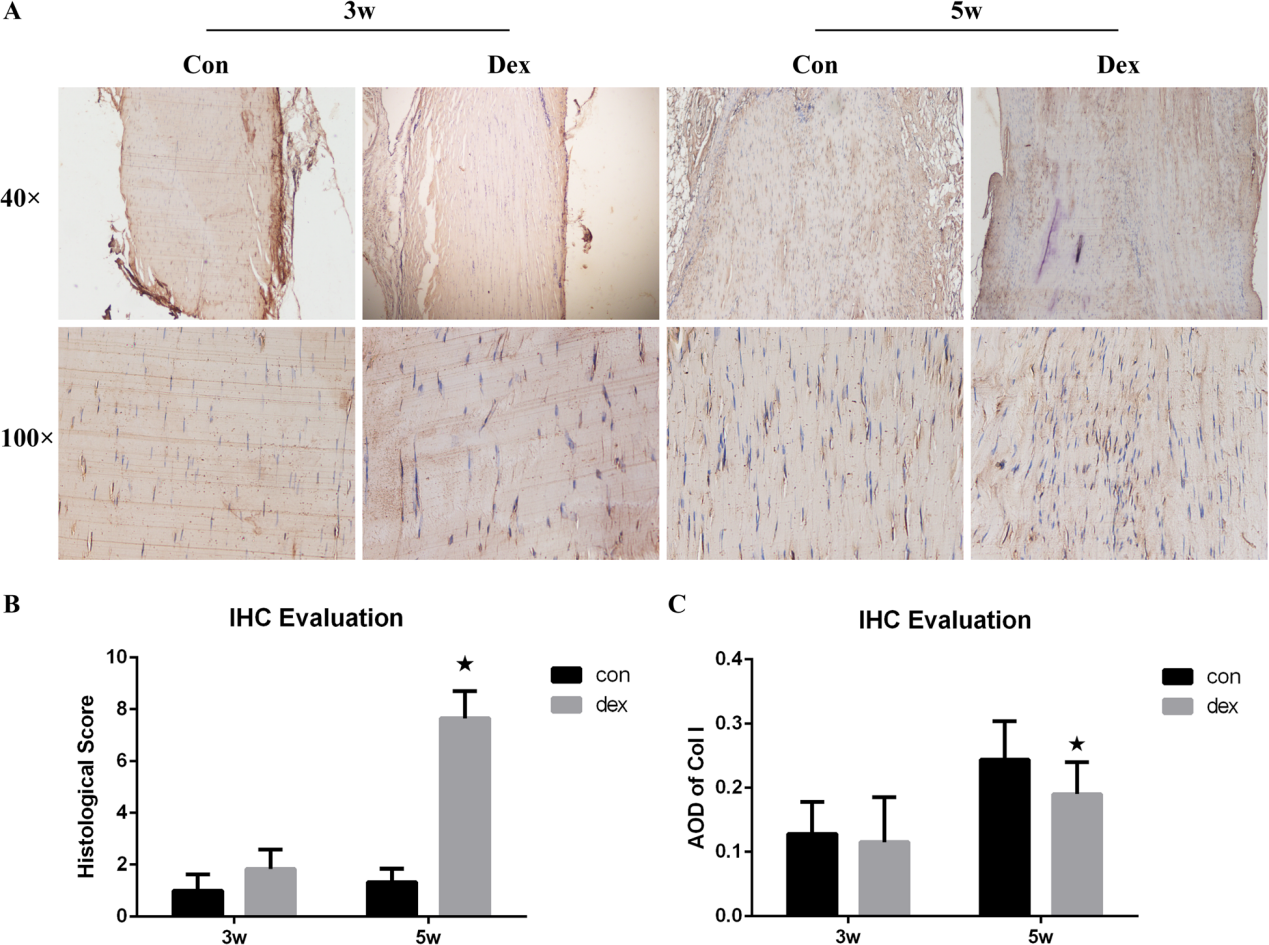
**a** Histology of rat Achilles tendon. The yellow bands represent type I collagen. **b** The histological score of immunohistochemical evaluation (IHC). **c** The average optimal density (AOD) of type I collagen expressed in rat Achilles tendons

To identify the effect of Dex on rat tendons, we observed changes in type I collagen expression at 3 and 5 weeks in Dex and control groups (Fig. 4a). The general pattern in the Dex group was the same as that for cells harvested from patients. Histological examination of tissue samples revealed that type I collagen of the Dex group was arranged irregularly and was curled and disordered compared with that of the control group. The overall collagen staining intensity in the field of view was also lower than that of the control group. The arrangement became substantially worse at 5 weeks. The histological score of tissue and AOD of type I collagen was showed in Fig. 4b and c.

**Fig. 5:**
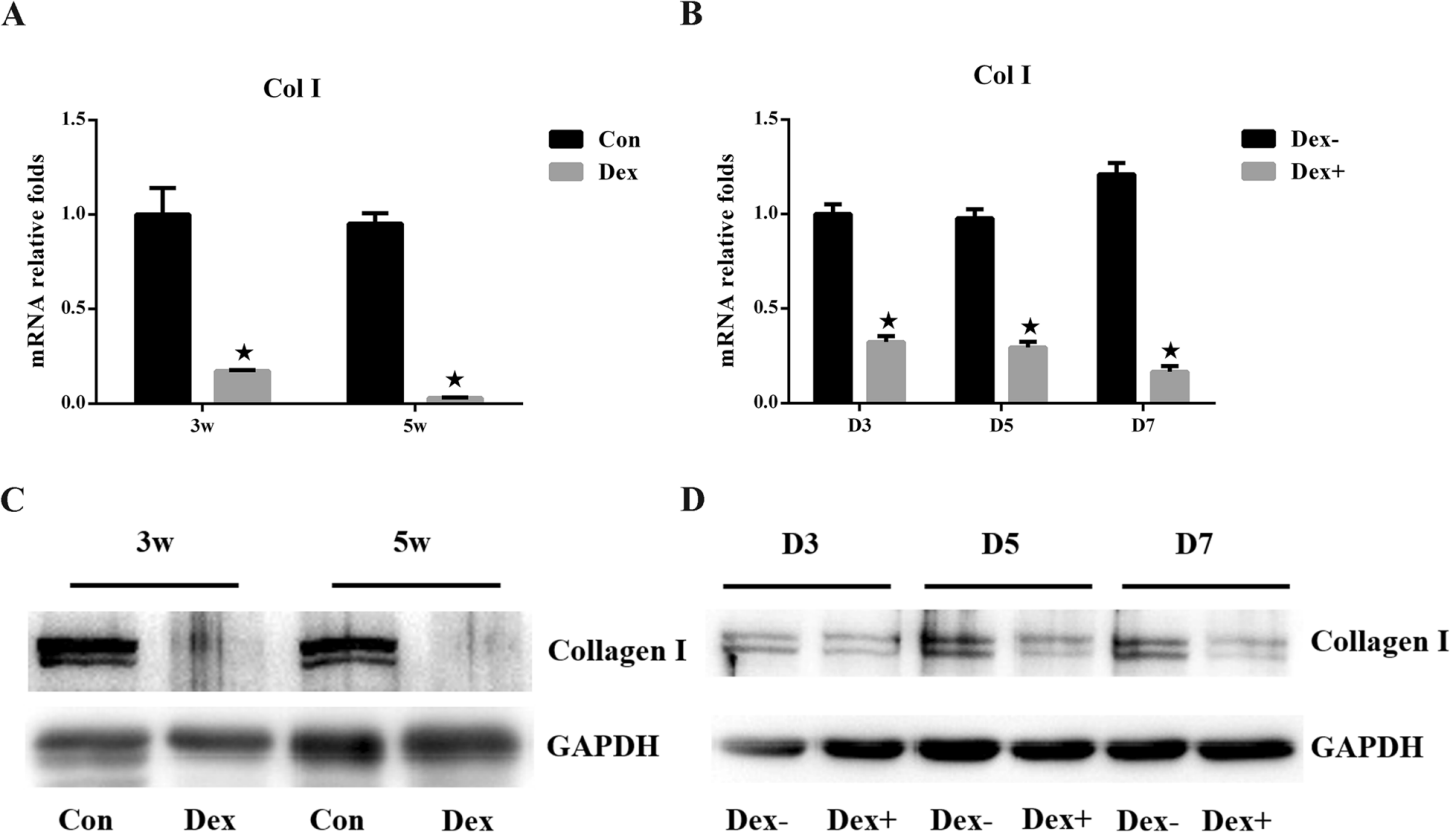
mRNA and protein expression of type I collagen in rat Achilles tendons tissues after 3 and 5 weeks (**a**) and (**c**). mRNA and protein expression of type I collagen in rat tenocytes at 3, 5, and 7 days (**b**) and (**d**). Relative expression levels were normalized to *GAPDH*. The asterisk represents a significant change between the two groups

Tissue samples were harvested at 3 and 5 weeks, and tenocytes were collected at day 3, day 5, and day 7 of culture. Results from qRT-PCR and western blotting are shown in Fig. 5. As the duration of Dex use increased, the expression level of type I collagen decreased, consistent with the previously described results. The identification of rat tenocytes is shown in Supplementary Figure 1.

### Tendon length

**Fig. 6:**
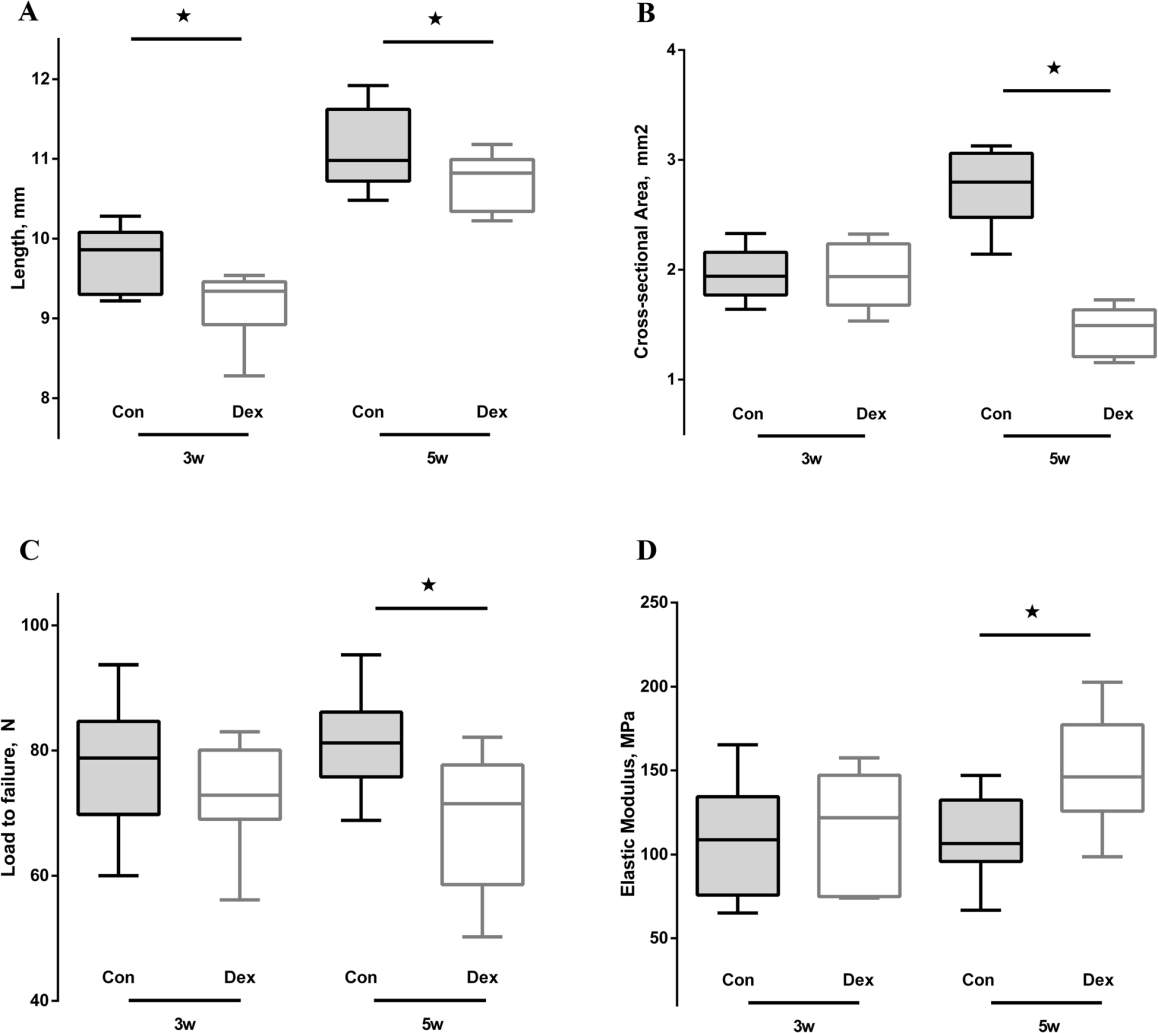
Mechanical testing of rat Achilles tendons. Control group (gray boxes); Dex group (white boxes); x-axis, 3 and 5 weeks. The line in the boxes represents the median, with the lower and upper ends of the boxes representing the 1st and 3rd quartiles, respectively. The asterisk represents a significant difference between the two groups

The tendon length significantly changed in both Dex groups. It was substantially shorter in the Dex group than in the control group at 3 weeks (Dex group, 9.18 ± 0.41 mm; control group, 9.72 ± 0.40 mm; *P* = 0.011) and 5 weeks (Dex group, 10.71 ± 0.34 mm; control group, 11.13 ± 0.50 mm; *P* = 0.050) (Fig. 6a).

### Cross-sectional area

The cross-sectional areas in the Dex group (1.95 ± 0.24 mm^2^) and control group (1.93 ± 0.29 mm^2^) were comparable at 3 weeks (*P* = 0.915). However, the areas were significantly lower in the Dex group (1.44 ± 0.22 mm^2^) than in the control group (2.74 ± 0.34 mm^2^) at 5 weeks (*P* < 0.001) (Fig. 6b).

### Load to failure

There was no significant difference between the Dex group (73.29 ± 8.27 N) and control group (77.21 ± 10.23 N) at 3 weeks (*P* = 0.386). However, a significantly lower maximal load to failure was observed in the Dex group (68.87 ± 11.07 N) than in the control group (81.46 ± 7.62 N) at 5 weeks (*P* = 0.013) (Fig. 6c).

### Elastic modulus

The elastic modulus of tendons from rats treated with Dex was higher than the control group at both time points, with slightly higher values at 3 weeks (Dex group, 112.04 ± 36.56 MPa; control group, 106.16 ± 34.20 MPa; *P* = 0.729), but significantly higher values for at 5 weeks (Dex group, 151.45 ± 32.22 MPa; control group, 111.01 ± 25.13 MPa; *P* = 0.010). The elastic modulus was significantly higher at 5 weeks than at 3 weeks in the Dex group (*P* = 0.027) (Fig. 6d).

## Discussion

In this study, we observed that samples from patients receiving long-term treatment of Dex showed downregulation and irregular arrangement of type I collagen. Experiments on human tenocytes showed significant decreases in type I collagen expression when cells were cultured in media containing Dex. In addition, in order to exclude interference from the clinic, we explored this hypothesis in rats on the cellular and tissue level. The results showed that Dex had a significant influence on the expression of type I collagen, both after 3 weeks and 5 weeks, but had a minimal effect on histology after 3 weeks. From the above findings, we concluded that long-term treatment with Dex downregulated type I collagen expression.

According to the results of the mechanical testing, the load to failure and elastic modulus of rat Achilles tendons were altered by Dex treatment. In addition, the length and cross-sectional area of the tendons were reduced by Dex treatment. However, aside from the tendon length, other parameters were not significantly changed after 3 weeks of Dex treatment in rats, indicating that the short-term use of Dex has little effect on tendon mechanical properties. By contrast, after 5 weeks of Dex treatment, all measured parameters changed significantly. The mean values for length, cross-sectional area, and load to failure decreased, and the elastic modulus increased. The difference in tendon length and cross-sectional area may be explained by the effect of Dex on type I collagen expression. According to the results of qRT-PCR and western blot, Dex treatment downregulated collagen type I on both the RNA and protein level. This likely contributed to the abnormal synthesis of collagen type I, leading to narrower and shorter Achilles tendons. Moreover, the mechanical properties of tendons are related to the fibril diameter, and the size of collagen fibrils determines functional differences. Fibrils with larger diameters exhibit an increased density of intramolecular cross-links, and those with smaller diameters are more elastic and resistant to creep [27]. Silver et al. reported that the elastic modulus and ultimate tensile strength are more dependent on fibril length than on diameter [28]. Additionally, type III collagen is more flexible than type I collagen [29]. Taguchi et al. reported that systemic administration of GC lead to smaller collagen fibers [16]. Considering Taguchi’s findings, the histological and mechanical changes noted in our study may be explained by the change of type I collagen. Long-term treatment with Dex resulted in irregular arrangement of type I collagen and a reduction in content, leading to a substantial effect on the load to failure and elastic modulus. With an increased elastic modulus and decreased maximal load to failure, the Achilles tendon had an increased risk of rupture.

Similar studies on the influence of corticosteroid injections on tendon mechanical properties have yielded different outcomes. Some studies have shown no significant change after local injection [30, 31], while others have reported the opposite. Haraldsson et al. proved that corticosteroid injection reduces the tensile strength of isolated collagen fascicles [32]. Mikolyzk et al. used a rat model and found that a single corticosteroid dose has significant short-term effects on the biomechanical properties of both injured and uninjured rotator cuff tendons [33]. In our study, significant changes in the molecular, histological, and mechanical characteristics of Achilles tendon tissue were identified after 5 weeks of Dex treatment, while only slight changes in some mechanical parameters and histological findings were observed by 3 weeks. This suggests that Dex probably had an impact on type I collagen since the treatment began; however, the impact was not sufficient to significantly alter the phenotype. As the treatment duration increased, significant histological and mechanical changes were identified, which will have increased the risk of Achilles tendon rupture.

These results suggest that type I collagen in the Achilles tendon can be decreased by Dex, but the underlying mechanism is not yet fully elucidated. Previous research provides some insight. Tomoyuki observed an increased expression of matrix metalloproteinase (MMP)-3 in response to corticosteroids [34]. MMP-3 is a potent proteoglycan-degrading enzyme that plays a vital role in the degeneration of collagen and other structural components [35]. MMP-3 expression levels are unchanged in the synovial tissues of osteoarthritic joints after the intra-articular administration of steroids, indicating its unique characteristic in tendons [36]. Other latent MMPs can degrade the extracellular matrix indirectly. In human supraspinatus tendon cells, migration is inhibited, and the levels of MMP2, MMP8, MMP9, and MMP13 are reduced, while tissue inhibitor of metalloproteinases 1, which is an MMP inhibitor, is upregulated [14]. In addition to MMPs, scleraxis is a probable gene involved in the alteration of type I collagen [37]. Therefore, a complex regulatory mechanism may determine the ultimate changes. Further research should focus on determining the detailed mechanism.

Some patients receive GC to treat the pain associated with Achilles tendinopathy. Recent research has shown that chronic inflammation is a feature of Achilles tendinopathy [38, 39]. Some doctors insist on using Dex to alleviate symptoms, as GCs inhibit the progression of inflammation. Although the specific pathophysiological mechanisms underlying tendinopathy remain unknown, four main drug classes have been implicated in its exacerbation, including long-term GCs. The main targets of GC toxicity are the lower limb tendons, including the Achilles tendon [40]. Considering the histological changes resulting from tendinopathy, including significant fiber disorientation [41], it is possible that GCs accelerate the progress of tendinopathy by leading to the decrease in type I collagen. Moreover, inflammation plays a vital role in tendon healing, but excessive or persistence inflammation can be deleterious [42]. As GC can inhibit angiogenesis, worsen the inflammation of micrangium, and trigger coagulation [43], it may impair collagen synthesis and tendon healing due to micro damage.

However, our study has a few limitations. Firstly, we obtained samples from patients who suffered from RA. The disease, as well as other factors, may cause structural damage to tendons, but the underlying mechanisms are not clear [44]. We performed an animal experiment to exclude these effects. However, we cannot conclusively exclude the effects of the disease. Secondly, the in vitro model of cultured human tendon cells only shows the short-term effect but not the long-term effect of Dex treatment. The difference in species and dose may also influence the findings. Although we used DMSO to help dissolve Dex to form a solution, the precipitation of Dex may still have occurred during in vivo and in vitro experiments, potentially affecting the conclusions regarding clinical applications. Moreover, the mechanism by which GC decreases type I collagen in the Achilles tendon remains to be fully elucidated.

## Conclusion

In conclusion, Dex downregulates type I collagen expression in Achilles tendons, resulting in tendon dysfunction and an increased risk of rupture. Dex, whether administered orally or by injection, requires an evaluation of the balance between the temporary positive responses and potential long-term side effects. Further research is needed to determine the precise mechanisms underlying the observed effects.

## Supplementary information

**Additional file 1: Figure S1.** The identification of rat tenocytes. Collagen type I and vimentin were positively expressed, and Collagen type III was negatively expressed.

## Data Availability

Not applicable.

## Abbreviations

AOD: Average optimal density
Dex: Dexamethasone
DMEM: Dulbecco’s modified Eagle’s medium
DMSO: Dimethyl sulfoxide
GC: Glucocorticoids
MMP: Matrix metalloproteinase
RA: Rheumatoid arthritis
