# Supplementary figures and images for: Downregulation of type I collagen expression in the Achilles tendon by dexamethasone: a controlled laboratory study
Source: J Orthop Surg Res. 2020 Feb 24;15:70. doi: 10.1186/s13018-020-01602-z (PMC7038574; doi:10.1186/s13018-020-01602-z)

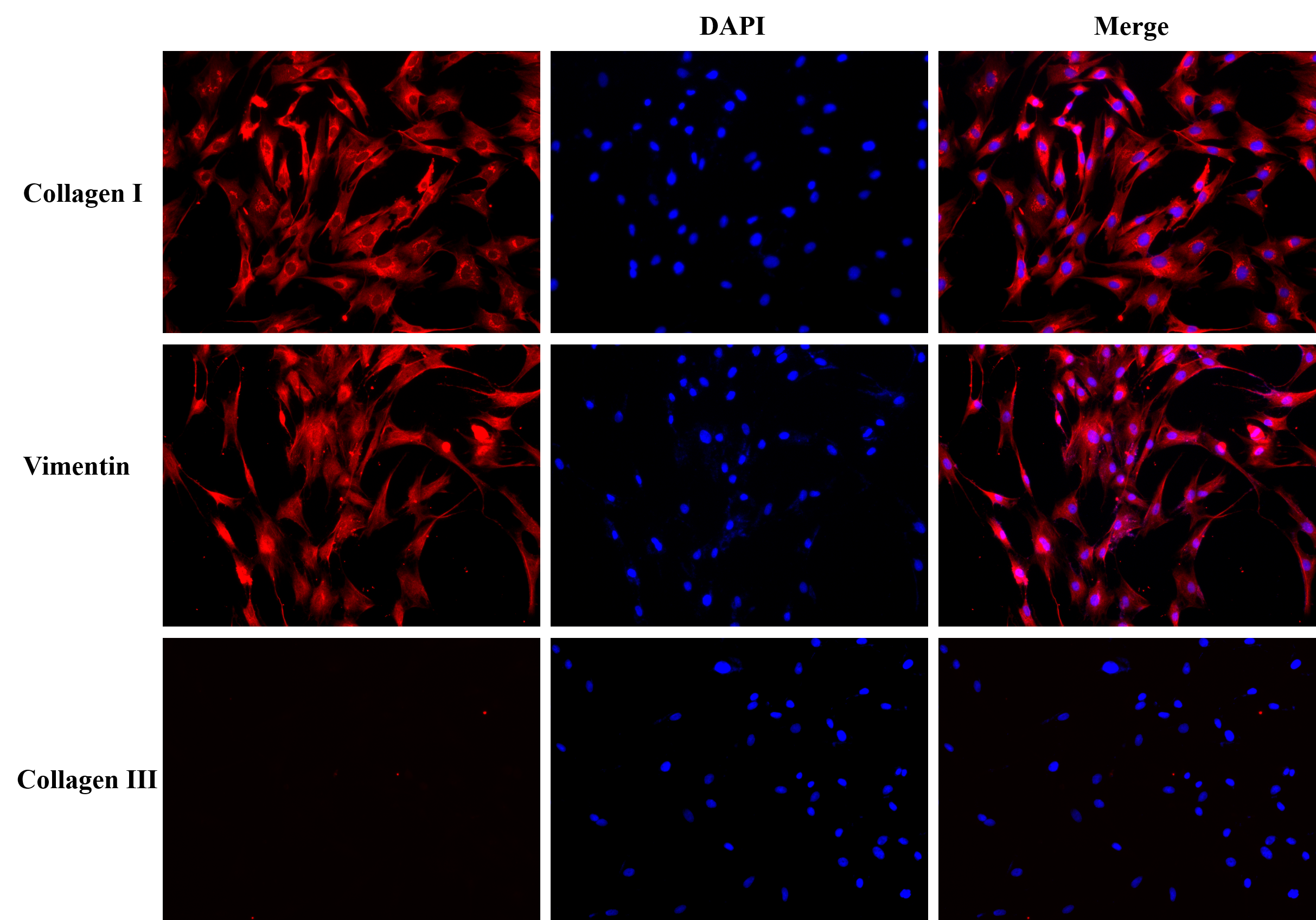

Supplement: Supplementary file 1 — Additional file 1: Figure S1. The identification of rat tenocytes. Collagen type I and vimentin were positively expressed, and Collagen type III was negatively expressed. [file 13018_2020_1602_MOESM1_ESM.tif]
